# Blurring emotional memories using eye movements: individual differences and speed of eye movements

**DOI:** 10.3402/ejpt.v7.29476

**Published:** 2016-07-04

**Authors:** Kevin van Schie, Suzanne C. van Veen, Iris M. Engelhard, Irene Klugkist, Marcel A. van den Hout

**Affiliations:** 1 Clinical Psychology, Faculty of Social and Behavioural Sciences, Utrecht University, Utrecht, The Netherlands; 2 Methodology and Statistics, Faculty of Social and Behavioural Sciences, Utrecht University, Utrecht, The Netherlands; 3 Research Methodology, Measurement and Data Analysis, Behavioral Sciences, Twente University, Enschede, The Netherlands

**Keywords:** Visual imagery, dual taxation, working memory capacity, EMDR

## Abstract

**Background:**

In eye movement desensitization and reprocessing (EMDR), patients make eye movements (EM) while recalling traumatic memories. Making EM taxes working memory (WM), which leaves less resources available for imagery of the memory. This reduces memory vividness and emotionality during future recalls. WM theory predicts that individuals with small working memory capacities (WMCs) benefit more from low levels of taxing (i.e., slow EM) whereas individuals with large WMC benefit more from high levels of taxing (i.e., fast EM).

**Objective:**

We experimentally examined and tested four prespecified hypotheses regarding the role of WMC and EM speed in reducing emotionality and vividness ratings: 1) EM—regardless of WMC and EM speed—are more effective compared to no dual task, 2) increasing EM speed only affects the decrease in memory ratings irrespective of WMC, 3) low-WMC individuals—compared to high-WMC individuals—benefit more from making either type of EM, 4) the EM intervention is most effective when—as predicted by WM theory—EM are adjusted to WMC.

**Method:**

Undergraduates with low (*n*=31) or high (*n*=35) WMC recalled three emotional memories and rated vividness and emotionality before and after each condition (recall only, recall + slow EM, and recall + fast EM).

**Results:**

Contrary to the theory, the data do not support the hypothesis that EM speed should be adjusted to WMC (hypothesis 4). However, the data show that a dual task in general is more effective in reducing memory ratings than no dual task (hypothesis 1), and that a more cognitively demanding dual task increases the intervention's effectiveness (hypothesis 2).

**Conclusions:**

Although adjusting EM speed to an individual's WMC seems a straightforward clinical implication, the data do not show any indication that such a titration is helpful.

Mental imagery allows us to think about past or anticipated events and is a powerful process with which we can reexperience and recombine perceptual information from memory (Kosslyn, Ganis, & Thompson, 2001). However, at times, it becomes maladaptive, for instance, when unwanted memories of upsetting life events come to mind. Intrusive, recurrent memories are core symptoms of posttraumatic stress disorder (PTSD) but can also occur in other psychiatric disorders, including obsessive–compulsive disorder, depression, body dysmorphic disorder, and several phobias (Hackmann & Holmes, 2004). These images can be past or future-oriented (e.g., Engelhard, Van den Hout, Dek, et al., 2011).

In clinical practice, cognitive behavioral therapy is often used to reduce intrusive imagery in posttraumatic stress disorder (Deacon & Abramowitz, 2004). It includes techniques that encourage patients to repeatedly relive these images (i.e., imaginal exposure) or to confront the feared object or situation in real life for prolonged periods of time (i.e., *in vivo* exposure; Rothbaum, Meadows, Resick, & Foy, 2000). However, patients with PTSD may be reluctant to be exposed to their feared images for longer periods of time (Arntz, Tiesema, & Kindt, 2007).

A different technique for manipulating image vividness and emotionality is used in eye movement desensitization and reprocessing (EMDR). In EMDR, patients make eye movements (EM) while they simultaneously recall traumatic memories. Research has shown that making EM not only reduces self-reported ratings of vividness and/or emotionality of unpleasant autobiographical memories (Engelhard, Van Uijen, & Van den Hout, 2010; Kavanagh, Freese, Andrade, & May, 2001; Maxfield, Melnyk, & Hayman, 2008; Van den Hout, Eidhof, Verboom, Littel, & Engelhard, 2014; for a meta-analysis of patient studies and analog studies, see Lee & Cuijpers, 2013) but also reduces the vividness and emotionality of imagined feared future events (i.e., flash-forwards; Engelhard, Van den Hout, Dek, et al., 2011; Engelhard, Van den Hout, Janssen, & Van der Beek, 2010). Furthermore, other secondary tasks besides EM also reduce image vividness and/or emotionality, including drawing a complex figure (Gunter & Bodner, 2008), playing Tetris (Engelhard, Van Uijen, et al., 2010), arithmetic (Engelhard, Van den Hout, & Smeets, 2011; Van den Hout et al., 2010), and complex tapping (Andrade, Kavanagh, & Baddeley, 1997).

How secondary tasks reduce vividness and emotionality of mental images can be conveniently explained by the interplay of dual taxation of working memory (WM; e.g., Andrade et al., 1997; Gunter & Bodner, 2008) and destabilization induced by memory reactivation (Lewis, 1979). A previously consolidated memory that is recalled (i.e., reactivated) can become labile and sensitive to disruption. When at the same time an individual performs a secondary task (e.g., making EM) dual taxation of WM takes place. Both tasks compete for limited WM resources and therefore the distressing memory cannot be retrieved completely (i.e., gets blurred). It is suggested that as a consequence of its temporary labile state the blurred memory reconsolidates after competition, and the reconsolidated blurred memory will be retrieved during future recalls (see Van den Hout & Engelhard, 2012).

WM theory predicts that the effectiveness of dual taxation depends on an individual's working memory capacity (WMC). For competition to occur, it is necessary that both tasks (mental image activation and secondary task) are sufficiently taxing. Individuals with a large WMC may be able to perform memory recall and a secondary task simultaneously without much competition between these tasks. As a consequence, they may experience fewer benefits (i.e., less blurring) from performing the dual task, compared to individuals with a relatively small WMC. Findings from Gunter and Bodner (2008, experiment 3) suggest that this is indeed the case: WMC—as measured with the automated reading span—correlated negatively with decreases in self-reported memory vividness/emotionality as a consequence of dual taxation. Other similar correlational findings support this (Engelhard, Van Uijen, et al., 2010; Van den Hout et al., 2010; Van den Hout, Engelhard, Beetsma, et al., 2011). Furthermore, the theory predicts that the dual task should not be too easy (this leaves too much capacity for the memory) or too hard (then the memory can hardly be recalled); the optimal load lies in between. There is preliminary evidence for this inverted U-curve shape: mild and moderately taxing of WM resulted in larger drops in emotionality ratings compared to little or extreme taxing (Engelhard, Van den Hout, & Smeets, 2011). This suggests that dual taxation of WM would be more effective if the degree of taxing is adjusted to an individual's WMC. More specifically, the theory predicts the presence of inter-individual differences; individuals with a relatively small WMC benefit more from relatively low levels of taxing and individuals with a relatively large WMC benefit more from relatively high levels of taxing. An interaction between WMC and WM taxation has obvious clinical implications. It would suggest that, in clinical practice, the degree of WM taxation (i.e., speed of EM) should be adjusted to the WMC of the patient treated. This is especially relevant because PTSD has been linked with poor performance on WMC measures (e.g., Samuelson et al., 2006). Since tasks measuring WMC are widely used and validated (Conway et al., 2005), it would be possible to determine a patient's individual WMC before the dual task intervention in EMDR is started.

Although WM theory predicts increased effectiveness when dual taxation is adjusted to individual differences, this might not necessarily be the case as suggested by an analogous study investigating intra-individual differences in memory vividness. Van Veen et al. (2015) inferred from WM theory that the effectiveness of dual taxation depends on intra-individual differences in memory vividness. They tested whether emotionality ratings of highly vivid memories (i.e., memories that are more taxing on WM) are more effectively reduced by a highly taxing secondary task (i.e., fast EM), and emotionality ratings of memories low in image vividness are more effectively reduced by an easy dual task (i.e., slow EM). Inconsistent with this prediction, there was no interaction with memory vividness. Emotionality ratings were reduced only as a result of increased dual taxation of WM; a high-load dual task led to higher reductions compared with a low-load dual task. This is in accordance with an earlier study by Maxfield et al. (2008) that also showed fast EM were more effective than slow EM, and that those were more effective than a control condition in decreasing image vividness and emotionality ratings. Therefore, there may only be a gradual effect of increasing dual taxation without an effect of WMC or load of the dual task.

All in all, it is possible that individuals with a small WMC benefit more from dual taxation in general compared with individuals with a large WMC as frequently evidenced in correlational studies. Alternatively, WM theory predicts that taxation is more effective if it is adjusted to an individual's WMC or to memory vividness. Previous research, however, showed that increasing dual taxation of WM increases effectiveness, regardless of intra-individual differences in memory vividness. It is therefore possible that only the dual taxation of WM determines its effectiveness irrespective of inter-individual differences in WMC.

Therefore, we tested four prespecified hypotheses regarding the role of individual WMC and dual taxation in reducing emotionality and vividness ratings. Within each hypothesis, we expect emotionality and vividness to display similar patterns. Our first hypothesis tested if making EM—regardless of EM speed—is more effective in reducing memory ratings compared to a control condition. The second examined if the speed of making EM solely affects the decrease in memory ratings irrespective of WMC. The third tested if low-WMC individuals—compared with high-WMC individual—benefit more from making EM in general, and our final hypothesis examined if reductions in memory ratings are highest when EM are adjusted to an individual's specific WMC. All hypotheses are visually presented in Fig. 1 and exact hypothesis constraints can be found in the Appendix. We used an experimental design—in which we manipulated speed of EM (i.e., load of the dual task) and tested individuals with low and high WMC—and we used a Bayesian approach to critically test which of the hypotheses is most likely.

**Fig. 1:**
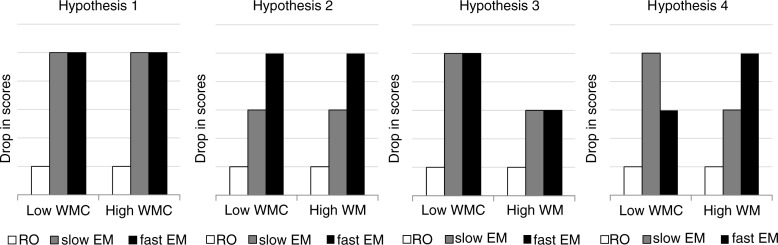
Visual representations of all four hypotheses for the low and high-WMC group after recall only (RO), slow EM, and fast EM.

## Method

### Participants

Prior to participation, undergraduate and graduate students from Utrecht University and the University of Applied Sciences (Hogeschool Utrecht) were screened for knowledge about the working mechanism of EMDR, participation in previous EMDR experiments, and medication intake that affected memory or concentration (*N*=166). Based on these exclusion criteria, 18 individuals were excluded from further participation. One hundred and forty-eight participants (*M*_*age*_=20.28, *SD*_*age*_=2.36; 64 males, 84 females) completed a WMC test for course credit or financial reimbursement. Thirteen of them were excluded because they failed to adequately perform the WM task (see below) or did not want to participate in the second part of the study, leaving a final sample of 135 participants. Via a tertiary split three groups were created. Individuals with the lowest and highest WMC were invited for the second part of our study and performed a dual-taxation task. Of the 90 participants invited, 37 low-WMC individuals and 38 high-WMC individuals were willing to participate further. After the dual-taxation task, six low-WMC individuals and three high-WMC individuals were excluded from the final data analysis, because they refused to make EM (*n*=1), failed to recall three emotional memories (*n=*2), or displayed emotionality and/or vividness scores that were extremely improbable and are indicative of task non-compliance (e.g., identical pre- and post-scores to two decimal places; *n=*6). Our final sample consisted of 31 and 35 participants, in the low and high-WMC groups, respectively (*M*_*age*_ *=*20.42, *SD*_*age*_=2.38; 25 males, 41 females). The Ethical Committee of the Faculty of Social and Behavioural Sciences of Utrecht University [FETC14-008 (Hout)] approved this study. Written informed consent was obtained from all participants.

### Materials & procedure

#### Automated reading span

Participants first completed the automated reading span (Conway et al., 2005; Daneman & Carpenter, 1980), which was used to assess WMC. Before the experimental trials, participants performed three practice sessions. In the first session, participants viewed two or three letters presented individually on screen for 1,000 ms. These letters were a subset of letters taken from 12 available letters: F, H, J, K, L, N, P, Q, R, S, T, and Y. Participants were instructed to remember the letters in the order in which they were presented. After a set of presented letters, participants were instructed to recall and indicate the letters they had just seen, in the same order, by clicking on letters presented in a 4×3 letter matrix. After each trial, the number of correctly selected letters was presented as feedback.

In the second session, participants read 15 sentences (e.g., “The young pencil kept his eyes closed until he was told to look.”) and decided after each sentence whether that sentence made sense by clicking a TRUE or FALSE box. After each decision, they received feedback on their accuracy. The program calculated each individual's mean decision time. This time around, plus 2.5 standard deviations served as maximum response latency for sentence evaluations in the experimental trials.

In the third session, participants practiced with both making sentence evaluations and remembering letters. Participants read a sentence and made an evaluation. This was immediately followed by one letter and the instruction to remember it. In this phase, trials comprised of two sentence–letter sequences (i.e., a set size of two). Thus, set size corresponded with the number of letters that had to be recalled at the end of a trial. After each trial, the letter matrix (cf. practice session 1) was shown, and participants recalled the letters they had just seen, in the same order. There were three practice trials with a set size of two.

Experimental trials were similar to trials in the third practice session except that the set size varied from three to seven, and set sizes were randomly presented. In total, 75 sentences and letters were presented in the experimental phase (three trials of each set size). Half of the sentences made sense, and half did not.

For scoring the automated reading span, we used partial-credit unit (PCU) scoring, in which credit is given to partly correct items as opposed to all-or-nothing unit scoring, where credit is only given to completely correct items (i.e., items where *all* letters were recalled in the correct order). That is, for a trial with a set size of four, two omissions followed by two correctly selected letters still constituted a score of 0.5 with PCU scoring. The PCU score expresses the proportion of correctly recalled letters within a trial averaged over all trials (see Conway et al., 2005, for a discussion on scoring in complex span tasks). Partial scores in the automated reading span show good test–retest reliability (*r*=0.82) and internal consistency (α=0.86–0.88), and the automated reading span correlates strongly with other complex span tasks measuring WMC (Conway et al., 2005; Redick et al., 2012).

#### Dual-taxation task

After a tertiary split on WMC, the highest and lowest groups performed a dual-taxation task. A participant was instructed to recall three negative autobiographical memories. These memories were rated on emotionality (0 *not unpleasant* to 100 *very unpleasant*). The emotionality score had to be in the range 50–90; if this was not the case, the participant was asked to recall a different memory. Next, the three memories were ranked from highest to lowest based on the participant's emotionality ratings. Then, for each memory, the participant selected the worst mental image, which served as that memory's “hotspot.” During this selection procedure, we counterbalanced which mental image was selected first, second, and third. For instance, some participants started with the memory ranked first, followed by the memory ranked second, and finally the memory ranked third (i.e., a 1–2–3 sequence). To avoid order effects, other counterbalancing sequences were used equally often (i.e., 1–3–2, 2–1–3, 2–3–1, 3–2–1, and 3–1–2). Next, the participant wrote down a label for each memory's “hotspot,” which served to refer to that specific mental image. After mental image selection, each of the participant's three mental images was assigned to one of the three conditions: recall+fast EM, recall+slow EM (henceforth called “fast EM” and “slow EM”), and recall only. So, a participant performed all conditions. Again, assignment to conditions depended on counterbalancing in a similar way as described for the selection phase. Counterbalancing assured that for all participants all three memories were equally often assigned to each of the conditions. The conditions (fast EM, slow EM, and recall only) were presented randomly to a participant. Before each condition, participants were presented with the label of their mental image and were instructed to recall the mental image. They then rated their memory on a visual analog scale (VAS) that ranged from 0 (*not vivid/unpleasant*) to 100 (*very vivid/unpleasant*). In each condition, participants recalled the memory for six intervals of 24 s separated by 10-s breaks (Engelhard et al., 2012; Van Veen et al., 2015). In both EM conditions, participants were seated approximately 45 cm from the computer screen and were instructed to recall the memory and to simultaneously follow a 20-pixel dot that moved horizontally with their eyes (600 pixel amplitude on a 1,280×1,024 pixel screen). For the slow EM condition, a dot moved with 0.8 Hz across the screen, and for the fast EM condition this was 1.2 Hz. A 1-Hz cycle corresponds with one left–right–left movement within 1 s. Van Veen et al. (2015) showed that these two speeds differed significantly in WM taxation. After each condition, participants again recalled the memory and rated it on vividness and emotionality using VASs. Groups were tested double blind.

### Data analysis

The hypotheses were evaluated using a Bayesian model selection criterion based on the Bayes factor (BF; Kass & Raftery, 1995) that was analyzed with the software BIEMS (see Mulder et al., 2009; Mulder, Hoijtink, & De Leeuw, 2012; Mulder, Hoijtink, & Klugkist, 2010). Contrary to null hypothesis significance testing, the Bayesian framework is not based on *p* values, dichotomous decisions (i.e., the result is significant or not), or the assumption that a null hypothesis is true. The Bayesian approach uses the observed data and computes support for each hypothesis given all constraints specified in each hypothesis. This approach can also be used to evaluate competing hypotheses. Thus, the calculated BF states the likelihood of a specified hypothesis. The program BIEMS specifically computes BFs for constrained hypotheses against the unconstrained hypothesis. A BF of 1 means that compared to an unconstrained model, the hypothesis receives equal support. BF>1 indicates that the hypothesis outperforms the unconstrained model, and BF<1 means the opposite.

## Results

### Automated reading span: working memory capacity scores

After the tertiary split, the group with the low-WMC group had a mean PCU score of 0.68, 95% CI [0.65, 0.71] and the high-WMC group had a mean score of 0.91, 95% CI [0.90, 0.92]. These mean scores correspond with the 30th and 75th percentile for the low and high group, respectively (Redick et al., 2012).

### Bayesian analysis on reductions in vividness and emotionality ratings

For each participant, a pre–post change score was calculated per condition for vividness and emotionality ratings; with higher scores indicating a greater pre–post drop.^1^ Bayesian analyses showed BFs of 3.02 and 4.02 for vividness and emotionality for hypothesis 1, 3.28 and 4.00 for hypothesis 2, 0.99 and 2.58 for hypothesis 3, and 0.11 and 1.08 for hypothesis 4. Overall, this shows that given the data, models 1 and 2 appear more likely than models 3 and 4 (see Table 1).

**Table 1:** Bayes factors for vividness and emotionality for all four hypotheses

	Hypothesis 1	Hypothesis 2	Hypothesis 3	Hypothesis 4
Vividness	3.02	3.28	0.99	0.11
Emotionality	4.02	4.00	2.58	1.08

### Observed reductions in vividness and emotionality ratings

**Fig. 2:**
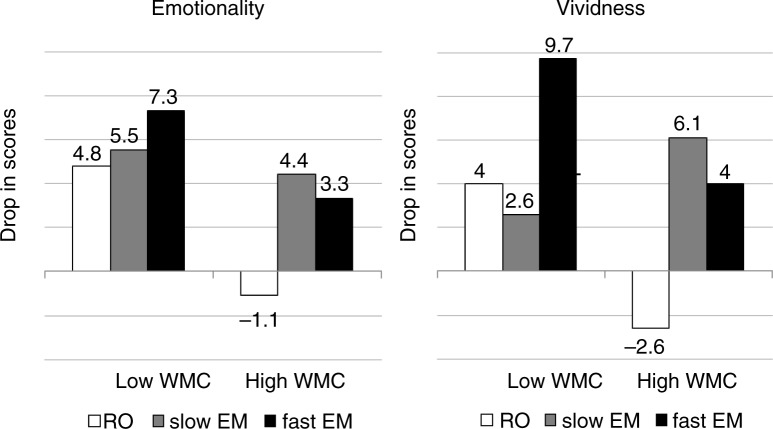
Observed reductions (pre–post difference scores) in emotionality and vividness for low and high-WMC group after recall only (RO), slow EM, and fast EM.

Figure 2 and Table 2 show that the observed data patterns are indeed moderately in line with hypothesis 1 and 2, but not with hypothesis 3 and 4 (note that in the hypotheses with between group comparisons, differences in recall only (i.e., control condition) are taken into account when comparing slow and fast EM conditions). However, the observed scores seem to display an unexpected pattern that was not hypothesized. In the low-WMC group, fast EM achieved the largest change scores, and in the high-WMC group, slow EM and fast EM seem to be equally effective in reducing memory ratings. It is also worth noting that the low and high-WMC group recall only conditions differ substantially: the low-WMC group showed a decrease in scores, while the high-WMC group showed an increase.

**Table 2:** Mean raw scores and standard deviations on emotionality and vividness before and recall only (RO), slow EM, and fast EM for the low and high-WMC group

	Emotionality			
	
	Low WMC		High WMC	
		
	Pre	Post	Pre	Post

Fast EM	68.87 (14.40)	61.59 (22.05)	68.16 (16.93)	64.48 (17.90)
Slow EM	71.24 (16.37)	65.62 (21.93)	70.74 (17.47)	66.31 (18.09)
RO	76.32 (16.03)	71.52 (16.60)	71.19 (16.44)	72.17 (16.72)
	Vividness			
	
	Low WMC		High WMC	
		
	Pre	Post	Pre	Post

Fast EM	73.25 (22.42)	63.51 (26.03)	71.37 (20.51)	67.38 (20.05)
Slow EM	71.22 (23.42)	68.66 (15.09)	73.78 (20.26)	68.03 (20.57)
RO	80.22 (14.95)	76.11 (20.27)	75.96 (17.55)	78.48 (16.03)

*Note*. These scores are not corrected for outliers. As a result, there are small deviations with the difference scores that were used for the analyses; WMC=Working Memory Capacity.

## Discussion

The aim of this study was to test if and how inter-individual differences in WMC affect self-reported memory ratings after recall of an emotional memory under different dual taxation conditions. Using a Bayesian approach, we compared four hypotheses, which specified the relation between WMC and WM taxation. Two of these hypotheses (hypothesis 1 and 2) were equally supported by the data. The data show that a dual task in general is more effective than no dual task, and that effectiveness increases with more cognitively demanding dual taxation. Moreover, it seems that individuals with smaller WMC do not benefit consistently from dual taxation compared with individuals with larger WMC (hypothesis 3), and that the hypothesis about adjusting WM taxation to WMC—as predicted by WM theory—(hypothesis 4) received very little support from the data and was therefore the least likely of all hypotheses. Unexpectedly, as was evident from the observed reductions, the low-WMC group showed the largest decrease after a high load dual task, while the high-WMC group showed similar decreases in memory ratings after either type of dual task.

The finding that an EM dual task in general (i.e., slow or fast EM) works better than no dual task (i.e., recall only)—as stated in hypothesis 1—joins a corpus of data showing that EM can be used as an effective dual task (see Lee & Cuijpers, 2013), and that any dual task that taxes WM is effective in reducing memory ratings of vividness and/or emotionality (e.g., Andrade et al., 1997; Engelhard, Van den Hout, & Smeets, 2011; Engelhard, Van Uijen, et al., 2010; Van den Hout et al., 2010). Evidently, competition for WM resources between a dual task and recall makes memories less vivid and less emotional during future recalls. Moreover, our study nicely fits with two other studies that have shown that a more cognitively demanding dual task results in larger decreases in memory ratings, and that different speeds of EM can be used to tax WM differentially (Maxfield et al., 2008; Van Veen et al., 2015). Our study replicates these findings and shows that a higher speed of EM leads to higher drops in memory ratings, as was evidenced by the support from the Bayes factor for hypothesis 2. The evidence in favor of hypothesis 3 was not only considerably smaller compared to hypothesis 1 or 2, but it was also mixed. The discrepancy between Bayes factors for emotionality and vividness makes interpretation of these results difficult. However, a change in emotionality, but not in vividness or vice versa, is not unique (e.g., Andrade et al., 1997; Engelhard, Van Uijen, et al., 2010; Maxfield et al., 2008). It is currently still unclear why the evidence is not consistent for both measures.

Though we found larger drops in vividness and emotionality after more cognitively taxing dual tasks using *digitalized* EM, these effects most likely translate well to clinical practice. Meta-analysis has shown that comparable effects have been found for digitalized EM and therapist-driven EM (Lee & Cuijpers, 2013). Moreover, from a theoretical point of view, the method of application is irrelevant as long as it sufficiently taxes WM. Indeed, other secondary tasks besides EM also reduce image vividness and/or emotionality, such as complex spatial tapping, playing Tetris, and mental arithmetic (Andrade et al., 1997; Engelhard, Van Uijen, et al., 2010; Van den Hout et al., 2010). Therefore, any intervention that sufficiently taxes WM should be effective.

The question remains why we did not find an interaction of WMC and WM taxation that was hypothesized based on WM theory (hypothesis 4). Possibly, the range of WMC of the university students recruited here was low and the tertiary split may not have resulted in between group differences that were sufficiently large. This nevertheless seems unlikely, because even in comparable student samples inverse correlations have been found between WMC and memory ratings after dual taxation (Gunter & Bodner, 2008; Engelhard, Van Uijen, et al., 2010; Van den Hout et al., 2010; Van den Hout, Engelhard, Beetsma, et al., 2011). Moreover, the average WMC scores for the low and high group corresponded with the 30th and 75th percentiles, respectively (Redick et al., 2012), which show that our groups differed meaningfully.

WM theory clearly predicted that inter-individual differences in WMC and different conditions of WM taxation should interact (Engelhard, Van den Hout, & Smeets, 2011; Gunter & Bodner, 2008). However, this study shows that there is little evidence that WM taxation is more effective when the degree of taxing is adjusted to interindividual differences in WMC. This is contradictory to studies suggesting an inverted U-curve in terms of WM taxation (Engelhard, Van den Hout, & Smeets, 2011; Gunter & Bodner, 2008) or to correlation studies (e.g., Gunter & Bodner, 2008; Van den Hout et al., 2010; Van den Hout, Engelhard, Beetsma, et al., 2011) that show that a standard speed of EM is most beneficial for individuals with smaller WMC, and that by extension higher speeds should be used for individuals with large WMC. The inconsistency in our study is not isolated but joins another found in Van Veen et al. (2015). They showed that intra-individual differences in memory vividness do not interact with WM taxation, though this was also hypothesized based on WM theory. The fact that in our study there was no trace of evidence suggesting an interaction between WMC and WM taxation serves as an anomaly for WM theory.

Still, the observed data show patterns that may be reconciled with WM theory. We found that for the low-WMC group, fast EM outperformed the other conditions, while for the high-WMC group either EM condition outperformed a no dual task control. Small reductions after slow and fast EM for the high-WMC group may be the result of a limitation of the amount of WM taxation from EM as a dual task for this group specifically. It is imaginable that fast EM in the study by Maxfield et al. (2008) and Van Veen et al. (2015)—and in our experiment—were the most taxing given the constraints determined by our muscular system, but that for the high-WMC group it may not have been cognitively taxing enough to achieve substantial drops in memory ratings. This problem of too little taxation for high-WMC individuals could be circumvented by increasing taxation by adding another task to EM that increases WM taxation as a whole, such as arithmetic (Engelhard, Van den Hout, & Smeets, 2011; Van den Hout et al., 2010; Van den Hout, Engelhard, Rijkeboer, et al., 2011). Alternatively, EM could be substituted by this task altogether to avoid constraints associated with making EM for prolonged periods of time. Arithmetic would be ideal because several factors of calculations can be manipulated to vary WM taxation (e.g., required operations or problem complexity; DeStefano & LeFevre, 2004).

In sum, we found that the data are in line with some predictions from WM theory: a dual task in general was effective in reducing memory ratings than no dual task, and a more cognitively demanding dual task increased the interventions’ effectiveness. However, differently taxing dual tasks did not interact with differences in WMC as hypothesized. This anomaly suggests that titration based on WMC does not increase the effectiveness of the dual task intervention. Adjusting WM load (i.e., EM speed) to the WMC of individual patients appears a straightforward clinical implication from WM theory for treatment with EMDR, but the data do not show any indication that such a titration is helpful. Based on our results and a study by Van Veen et al. (2015), the only clinical implication that follows is that increasing speed of EM, increases the intervention's effectiveness.

## Footnotes

1 Changed scores with more than three SD from the group mean were corrected to a score with three SD from the group mean. In total, there were 396 difference scores of which 6 were corrected.

## Appendix

**Table:** Hypothesis constraints for vividness and emotionality difference scores for each of the four hypotheses

Hypothesis	Constraints
Hypothesis 1	Both WMC groups: (Fast EM, Slow EM)>Recall Only
Hypothesis 2	Both WMC groups: Fast EM>Slow EM>Recall Only
Hypothesis 3	Both WMC groups: (Fast EM, Slow EM)>Recall Only
	(Fast EM_low_−Recall Only_low_)>(Fast EM_high_−Recall Only_high_)
	(Fast EM_low_−Recall Only_low_)>(Slow EM_high_−Recall Only_high_)
	(Slow EM_low_−Recall Only_low_)>(Fast EM_high_−Recall Only_high_)
	(Slow EM_low_−Recall Only_low_)>(Slow EM_high_−Recall Only_high_)
Hypothesis 4	Fast EM_high_>Slow EM_high_>Recall Only_high_
	Slow EM_low_>Fast EM_low_>Recall Only_low_
	(Slow EM_low_−Recall Only_low_)>(Slow EM_high_−Recall Only_high_)
	(Fast EM_high_−Recall Only_high_)>(Fast EM_low_−Recall Only_low_)

*Note*. WMC=Working Memory Capacity; Low=Low WMC; High=High WMC; EM=Eye Movements. A comma separating two conditions within parentheses indicates that the following inequality constraint (e.g.,<or >) applies to both conditions independently.
